# Contributions of plasmid p1AB5075-encoded antibiotic resistance genes to multidrug resistance of Acinetobacter baumannii AB5075

**DOI:** 10.1099/jmm.0.002189

**Published:** 2026-07-24

**Authors:** Orlaith Plunkett, Anna S. Ershova, Kristina Schauer, Carsten Kröger

**Affiliations:** 1 Department of Microbiology, School of Genetics & Microbiology, Moyne Institute of Preventive Medicine, Trinity College Dublin, Dublin 2, Ireland

**Keywords:** *Acinetobacter baumannii* AB5075, antibiotic resistance, p1AB5075, resistance island 2

## Abstract

Infections caused by multidrug-resistant *Acinetobacter baumannii* are considered a threat to human and animal health. The widely studied *A. baumannii* strain AB5075 displays a high degree of antibiotic resistance. In this study, we experimentally validated that antibiotic resistance is largely mediated by resistance genes located on plasmid p1AB5075. We used a p1AB5075-deficient AB5075 strain to assess individual contributions of p1AB5075-encoded antibiotic resistance genes by ectopically (over-)expressing each gene in the Δp1AB5075 background. By determining individual contributions of seven p1AB5075-encoded antibiotic resistance genes, we show individual and overlapping roles of genes for aminoglycoside resistance and uncover the importance of extended-spectrum *β*-lactamase *bla_GES-11_* for monobactam and cephalosporin resistance in *A. baumannii* AB5075. We discovered that aminoglycoside *N*-acetyltransferase *aaC(6′)-Ib3* (*aacA4*), which was previously shown to confer resistance to tobramycin, provides broad resistance to gentamicin, kanamycin, amikacin, streptomycin and tobramycin when overexpressed in *A. baumannii* AB5075. Because p1AB5075 is transferable to a wide range of environmental and clinical *A. baumannii* strains and non-*baumannii Acinetobacter* species, the relevance of our findings extends beyond *A. baumannii* AB5075.

## Introduction

Curing infections caused by multidrug-resistant (MDR) micro-organisms in humans and animals is one of the greatest medical challenges of the twenty-first century. Among the most problematic bacteria that require investment into research and development of new antibiotics or alternative treatment strategies are carbapenem-resistant *Acinetobacter baumannii* [1–3]. Infections with *A. baumannii* are increasingly difficult to clear because of a high degree of antimicrobial resistance (AMR), strain heterogeneity and reports of pan-drug-resistant strains [4–6]. Understanding the evolution of antimicrobial resistance and resistance mechanisms may inform the development of new drugs and better treatment regimes. Early isolates that have been extensively used to study *A. baumannii* biology were typically susceptible to antibiotics, including the widely studied strains *A. baumannii* ATCC17978 and ATCC19606 [7]. Contemporary *A. baumannii* strains, including the widely studied *A. baumannii* AB5075, are likely more suitable representatives of current infections and are often resistant to a large array of antibiotics [8].

In *A. baumannii* AB5075, many antibiotic resistance genes (ARGs) are located on plasmid p1AB5075 within ‘Resistance Island 2’ (RI-2), but some are also found on the chromosome (e.g. *blaOXA-23*) [8, 9]. The plasmid p1AB5075 is the largest of three plasmids in *A. baumannii* AB5075 (83,160 bp), which was shown to be transferable to other *A. baumannii* strains of environmental and clinical origin and to diverse non-*baumannii Acinetobacter* species [8–11]. The RI-2 locus is chiefly responsible for aminoglycoside (hetero-)resistance in *A. baumannii* AB5075, which is caused in part by a RecA-dependent resistance island amplification to up to 20–24 copies [12]. The RI-2 small RNA SrvS located upstream of *aadB* was also shown to be involved in virulent opaque (VIR-O) to avirulent translucent colony (AV-T) type switching, showing that p1AB5075-encoded genes are engaging in regulatory cross-talk between p1AB5075 and the chromosome [13]. RI-2 is mosaic and contains, among other ARGs, an extended-spectrum *β*-lactamase *bla*_GES-11_, which was first described in an *A. baumannii* strain isolated in France [14].

Despite their biological importance, spontaneous loss of large plasmids, including p1AB5075, has been documented for *A. baumannii* [5]. Plasmid pAB3 has been reported to have been lost in *A. baumannii* ATCC17978, which caused up-regulation of the Type-6 Secretion System [15, 16], and loss of p1AB5075 in *A. baumannii* AB5075 resulted in susceptibility to amikacin and tobramycin and reduced resistance to chloramphenicol [13, 17]. Here, we report loss of p1AB5075 in *A. baumannii* AB5075 independent of the published reports [13, 17]. We used the Δp1AB5075 genetic background to characterize the antibiotic resistance profile for the p1AB5075-deficient strain and genetically dissect the contributions of seven individual p1AB5075-encoded antibiotic resistance genes for high-level multidrug resistance.

## Methods

### Bacterial strains and general growth conditions

*A. baumannii* AB5075 and *Escherichia coli* TOP10 (Invitrogen) were maintained on lysogeny broth (Lennox, l-) agar plates (10 g l^−1^ tryptone, 5 g l^−1^ yeast extract, 5 g l^−1^ NaCl and 15 g l^−1^ agar) and grown overnight in liquid l-broth (10 g l^−1^ tryptone, 5 g l^−1^ yeast extract and 5 g l^−1^ NaCl) [8]. Media contained tetracycline (12 µg ml^−1^) when necessary to maintain plasmid pWH1266 [18]. Opaque (VIR-O subpopulation) colonies were used throughout the study.

### Strain and plasmid constructions

ARGs were amplified from purified genomic DNA of *A. baumannii* AB5075 using DNA oligonucleotides listed in Table S1 (available in the online Supplementary Material) with ~20 bp overhangs complementary to the pWH1266 backbone to enable seamless ligation-independent cloning extract (SLiCE)-mediated cloning [19]. PCR amplification of DNA was performed using Verify^™^ polymerase (PCR Biosystems). Plasmid isolations and PCR purifications were carried out according to the manufacturer’s protocol using EasyPure Plasmid MiniPrep and PCR Purification Kits (TransGen Biotech). SLiCE cloning procedure was used to insert ARGs into the PCR-amplified pWH1266 backbone [19]. All plasmid construction steps were carried out in *E. coli* TOP10 cells. To ensure efficient and equal translation of ARG mRNAs, the forward primer contained an artificial AGGAGG ribosome-binding site. The cloned genes were constitutively expressed from the *β*-lactamase (*blaTEM-1*) promoter of pWH1266 [18]. Plasmids were re-isolated from *E. coli*, their insert sequence verified by Sanger sequencing (Eurofins) and AB5075 was subsequently transformed with the plasmids using natural transformation or electroporation [20]. Whole plasmid sequencing of pWH1266 was conducted by Azenta/GENEWIZ (GENEWIZ Germany GmbH) using Oxford Nanopore Technology in a GridION with a FLO-MIN114 (R10.4.1) flow cell (GenBank accession no.: PV577797.1).

### Disk diffusion assays

All *A. baumannii* AB5075 strains were cultured overnight in cation-adjusted Mueller–Hinton broth (MH2, Merck/Millipore). Overnight cultures were adjusted to 0.5 McFarland standard, and each culture sample was swabbed thoroughly onto Mueller–Hinton agar plates using a sterile cotton swab. Plates were air-dried for 10 min before the addition of antimicrobial susceptibility discs (Oxoid). Antibiotic discs contained amikacin (AK, 30 µg), tobramycin (TOB, 10 µg), kanamycin (K, 30 µg), gentamicin (CN, 10 µg), trimethoprim–sulphamethoxazole (SXT, 25 µg), streptomycin (S, 25 µg), meropenem (MEM, 10 µg), doripenem (DOR, 10 µg), imipenem (IPM, 10 µg), vancomycin (VA, 30 µg), erythromycin (E, 15 µg), nitrofurantoin (F, 100 µg), tetracycline (TE, 30 µg), tigecycline (TGC, 15 µg), ciprofloxacin (CIP, 5 µg), cefepime (FEP, 30 µg), cefoperazone (CFP, 30 µg), ceftazidime (CAZ, 30 µg), chloramphenicol (C, 30 µg), aztreonam (ATM, 30 µg), oxacillin (OX, 1 µg), ticarcillin (TIC, 75 µg), cefoxitin (FOX, 30 µg) and piperacillin/tazobactam (TZP, 110 µg; piperacillin 104.3 µg, tazobactam 10.2 µg). Plates were incubated overnight at 37 °C before measuring the diameters of inhibition zones.

### Broth microdilution assay

Broth microdilution assays were performed as per the Clinical and Laboratory Standards Institute guidelines for *Acinetobacter* to determine the MIC for antibiotics. The assays were performed in 96-well plates. The standardized inoculum (0.5 McFarland) was prepared by direct colony suspension in sterile 0.9% (w/v) saline. The inoculum was then diluted in MH2 (cation-adjusted Mueller–Hinton broth, CAMHB) broth to obtain a final concentration of 5×10^5^ c.f.u. ml^−1^ in each well except for the negative control (MH2 medium without bacteria). Plates were incubated at 37 °C statically for 22 h before the plates were analysed visually. Antibiotics were sourced from the following suppliers: Imipenem (Acros Organics), meropenem (Thermo Scientific), doripenem (Thermo Scientific), cefepime (Sigma), cefotaxime (Melford), aztreonam (Sigma), tobramycin (Sigma), gentamicin (Roth), amikacin (Cayman Chemical Company), streptomycin (Sigma), kanamycin (Sigma), ciprofloxacin (Sigma), levofloxacin (Sigma), norfloxacin (Sigma), colistin (Sigma), tetracycline (Sigma), tigecycline (Sigma), erythromycin (Sigma) and colistin (Sigma).

### Growth experiments

Growth of bacterial strains was measured in a volume of 200 µl l-broth containing 12 µg ml^−1^ tetracycline for pWH1266 plasmid maintenance in 96-well plates. A single colony of each strain was resuspended in l-broth and the absorbance at 600 nm (A_600_) was normalized to 0.05 in 200 µl l-broth and incubated with orbital shaking at 37 °C and 282 cycles per minute in a BioTek Synergy HTX Multimode Reader. Every 20 min, the A_600_ was recorded for 24 h.

## Results

### Loss of p1AB5075 results in extensive susceptibility to multiple antibiotics

*A. baumannii* AB5075 is considered an MDR strain with increased virulence and a contemporary representative of *A. baumannii* infections. Because *A. baumannii*, and especially *A. baumannii* AB5075, shows a high degree of phenotypic and genotypic heterogeneity [5, 21–26], we whole-genome sequenced a number of *A. baumannii* AB5075 colonies after attempting to delete genes AB5075 by the suicide-vector method of Pokhrel *et al*. [27]. Serendipitously, this revealed that one of the sequenced colonies, where the wild-type genotype at the position of the attempted deletion was restored, had lost plasmid p1AB5075 but was otherwise wild-type – an event which had been independently reported before [17].

**Fig. 1.**
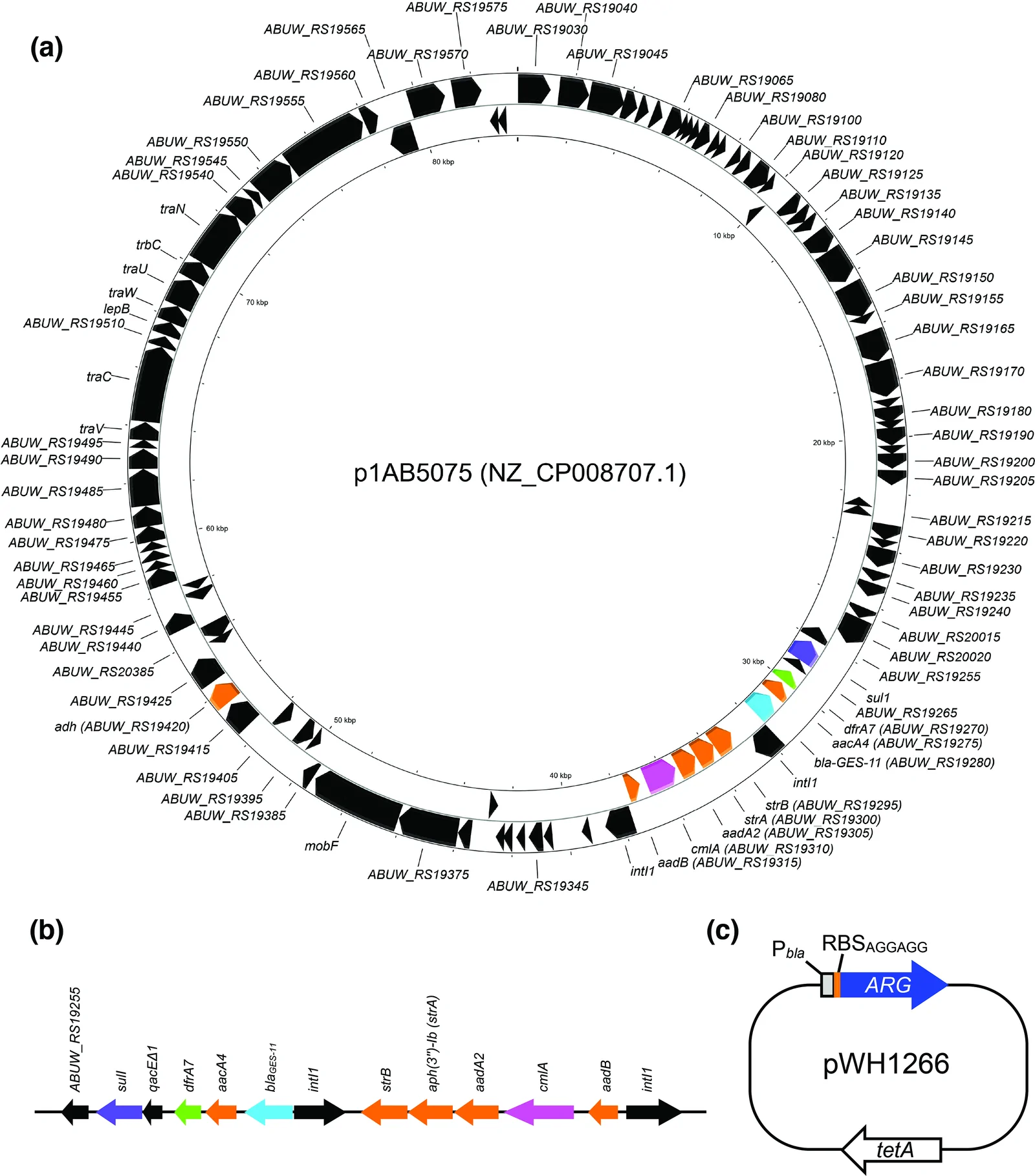
Map of *A. baumannii* AB5075 p1AB5075 (**a**), Resistance Island-2 (**b**) and complementation plasmid pWH1266 (**c**). (**a**) Aminoglycoside genes are highlighted in orange, chloramphenicol resistance gene *cmlA* in pink, sulphonamide resistance gene *sul1* in light green, beta-lactamase-encoding *bla*_GES-11_ in light blue and trimethoprim resistance gene *dfrA7* in purple. The map was created with Proksee [35]. (**b**) Linear depiction of p1AB5075-encoded Resistance Island-2. (**c**) Schematic map of the pWH1266 plasmid used for complementation. The *blaTEM-1* gene of pWH1266 was replaced by the ARG (blue) resulting from transcription of ARGs from the P*_blaTEM-1_* promoter (grey, P*_bla_*). All ARGs are translated using the same ribosome-binding site (orange, RBS_AGGAGG_). The *tetA* gene of pWH1266 confers resistance to tetracycline. The plasmid is not drawn to scale.

As p1AB5075 carries multiple antibiotic resistance genes as part of RI-2, we set out to characterize the antibiotic resistance profile of the Δp1AB5075 strain and to investigate the individual contributions of ARGs on p1AB5075, which so far, with the exception of *cmlA* contributing to chloramphenicol resistance, had not been experimentally verified gene-by-gene in *A. baumannii* AB5075. The predicted ARGs of p1AB5075 are shown in the context of p1AB5075 in Fig. 1, and their annotation is presented in Table 1. All ARGs except one, a predicted APH(3′)-VI family aminoglycoside O-phosphotransferase (*adh*/*ABUW_RS19420*), are located within RI-2 (Fig. 1A & B). Antibiotic disc diffusion assays were performed to assess antibiotic resistance profiles of wild-type AB5075 and the Δp1AB5075 mutant strain to a suite of antibiotics.

**Table 1.** Annotation of antibiotic resistance genes of p1AB5075

Gene	Locus ID	Annotation
*adh*	*ABUW_RS19420*	APH(3′)-VI family aminoglycoside O-phosphotransferase
*sul1*	*ABUW_RS19260*	Sulphonamide-resistant dihydropteroate synthase
*qacEΔ1*	*ABUW_RS19265*	Quaternary ammonium compound efflux SMR transporter QacE Δ1
*dfrA7*	*ABUW_RS19270*	Trimethoprim-resistant dihydrofolate reductase DfrA7
*aacA4*	*ABUW_RS19275*	Aminoglycoside *N*-acetyltransferase AAC(6′)-Ib3
*bla_GES-11_*	*ABUW_RS19280*	Extended-spectrum class A beta-lactamase GES-11
*strB*	*ABUW_RS19295*	APH(6)-I family aminoglycoside O-phosphotransferase
*aph(3'')-Ib* (*strA*)	*ABUW_RS19300*	Aminoglycoside O-phosphotransferase APH(3'')-Ib
*aadA2*	*ABUW_RS19305*	ANT(3″)-Ia family aminoglycoside nucleotidyltransferase AadA2
*cmlA*	*ABUW_RS19310*	CmlA family chloramphenicol efflux MFS transporter
*aadB*	*ABUW_RS19315*	Aminoglycoside nucleotidyltransferase ANT(2″)-Ia

**Fig. 2.**
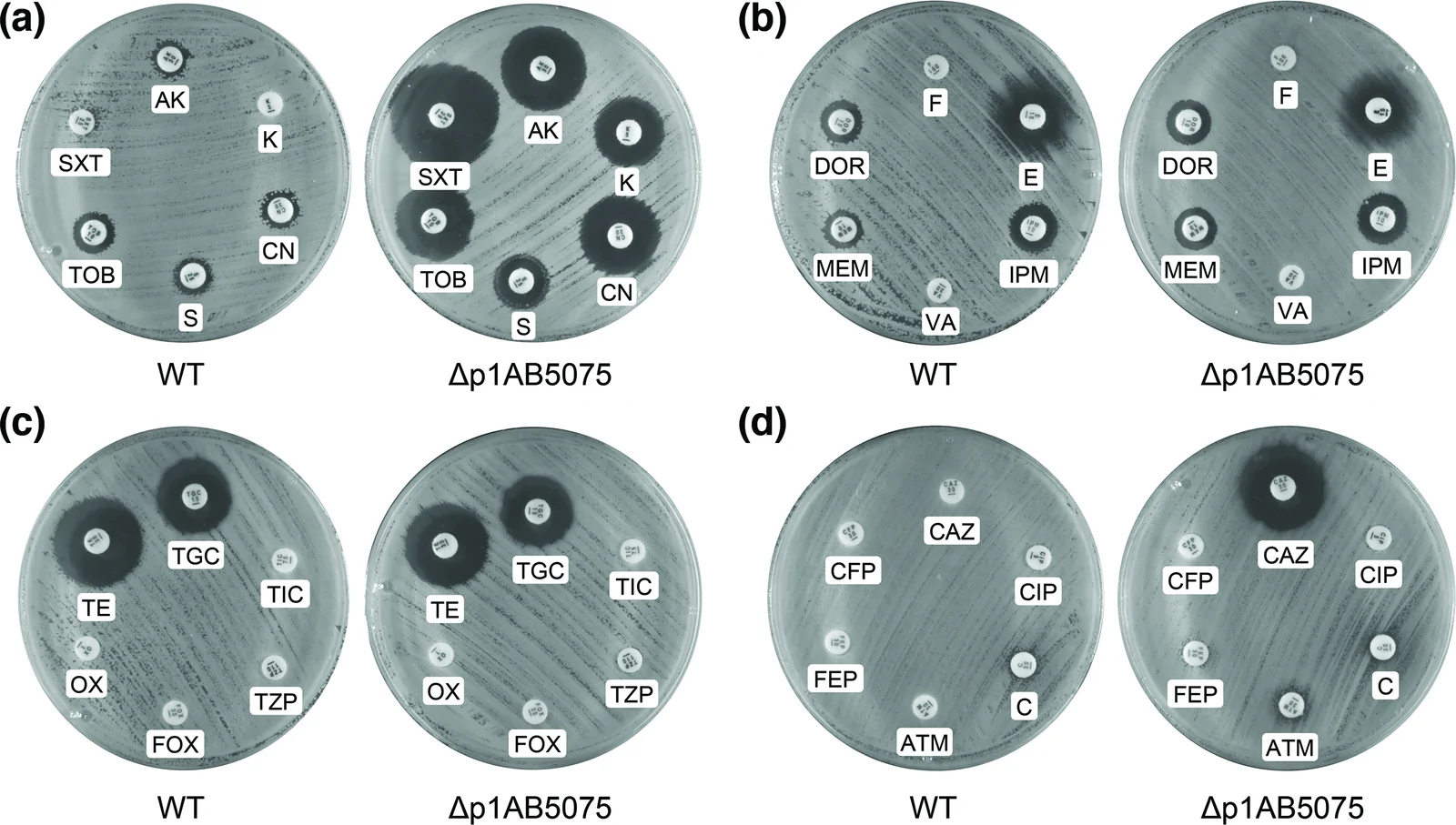
Antibiotic disc diffusion assays comparing *A. baumannii* AB5075 wild-type (WT) and Δp1AB5075 strains. MH2 agar plates were lawned with wild-type *A. baumannii* AB5075 and Δp1AB5075, and antibiotic-containing discs were placed on the agar surface. The plates were incubated for 24 h at 37 °C. (**a**) AK (amikacin 30 µg), SXT (trimethoprim–sulphamethoxazole 25 µg), TOB (tobramycin 10 µg), S (streptomycin 25 µg), CN (gentamicin 30 µg), K (kanamycin 5 µg), (**b**) nitrofurantoin (F 100 µg), D (doripenem 10 µg), MEM (meropenem 10 µg), VA (vancomycin 20 µg), IPM (imipenem 10 µg), E (erythromycin 15 µg), (**c**) TGC (tigecycline, 15 µg), TE (tetracycline, 30 µg), OX (oxacillin, 1 µg), FOX (cefoxitin, 30 µg), TZP (piperacillin/tazobactam, 110 µg), TIC (ticarcillin, 75 µg), (**d**) CAZ (ceftazidime, 30 µg), CFP (cefoperazone, 30 µg), FEP (cefepime, 30 µg), ATM (aztreonam, 30 µg), C (chloramphenicol, 30 µg) and CIP (ciprofloxacin, 5 µg).

The Δp1AB5075 mutant strain showed widespread increased susceptibility including to all tested aminoglycosides (amikacin, gentamicin, kanamycin, streptomycin and tobramycin), but also to trimethoprim/sulphamethoxazole, aztreonam (monobactam) and ceftazidime (cephalosporin) (Fig. 2, Table 2) highlighting the crucial role of p1AB5075 in multidrug resistance. No difference was observed to ciprofloxacin (fluoroquinolone), cefoperazone, cefepime (cephalosporins), doripenem, imipenem, meropenem (carbapenems), nitrofurantoin (nitrofuran), erythromycin (macrolide), vancomycin (glycopeptide), tetracycline, tigecycline (tetracyclines), cefoxitin (cephamycin), oxacillin, ticarcillin (*β*-lactams), chloramphenicol and the broad-spectrum *β*-lactam antibiotic/inhibitor combination piperacillin/tazobactam. We noted a difference in cephalosporin resistance, where wild-type and Δp1AB5075 strains were resistant to cefoperazone and cefepime but not ceftazidime, indicating that ceftazidime resistance is mediated by Δp1AB5075 (Fig. 2). Resistance to carbapenems did not change, which is mediated by a chromosomally encoded *blaOXA-23* gene [28]. Resistance to chloramphenicol was not altered either, likely due to the presence of additional chloramphenicol resistance genes *craA* and *cpxE* (*ABUW_0982*).

**Table 2.** Average of inhibition zones of AB5075 mutants measured in mm (*n*=3) determined by Kirby–Bauer disc diffusion assays

	Aminoglycoside					Tetracyclines		*β*-Lactam											Others					
	AK 30	CN 30	K 5	S 25	TOB 10	TE 30	TGC 15	IPM 10	MEM 10	DOR 10	CFP 30	CAZ 30	FEP 30	ATM 30	TIC 75	OX 1	FOX 30	TZP 110	CIP 5	C 30	SXT 25	F 100	E 15	VA 30
Wild-type	7	0	0	9	10	24	19	12	9	11	0	0	0	0	0	0	0	0	0	0	0	0	22	0
Δp1AB5075	21	20	17	16	18	22	17	13	10	12	0	19	0	7	0	0	0	0	0	7	25	0	20	0
Wild-type pWH1266	9.5	10	0	12	11	10	18	12	10	12	0	0	0	0	0	0	0	0	0	0	0	0	21	0
Δp1AB5075 pWH1266	24	21	19	15	18	10	19	12	9	12	0	17	18	8	0	0	0	0	0	7	26	0	19	0
Δp1AB5075 pWH1266-*aadB*	21	0	0	15	0	10	15	14	10	13	0	19	15	10	0	0	0	0	0	10	27	0	20	0
Δp1AB5075 pWH1266-*adh*	0	23	0	16	19	10	16	12	10	12	0	20	17	9	0	0	0	0	0	9	28	8	20	0
Δp1AB5075 pWH1266-*aacA4*	21	20	8	16	14	9	18	14	14	14	0	22	19	11	0	0	0	0	0	12	25	7	21	0
Δp1AB5075 pWH1266-*aadA2*	27	22	19	0	18	9	15	12	10	11	0	18	17	10	0	0	0	0	0	8	29	7	19	0
Δp1AB5075 pWH1266- *strA*	27	25	18	0	17	11	18	14	10	12	0	18	15	10	0	0	0	0	0	10	30	8	20	0
Δp1AB5075 pWH1266- *strB*	24	23	17	17	18	10	15	12	10	11	0	20	19	12	0	0	0	0	0	11	28	8	20	0
Δp1AB5075 pWH1266-*bla_GES-11_*	23	23	26	12	21	0	17	13	10	12	0	0	0	0	0	0	0	0	0	8	30	8	21	0

AK, amikacin; ATM, aztreonam; C, chloramphenicol; CAZ, ceftazidime; CFP, cefoperazone; CIP, ciprofloxacin; CN, gentamicin; DOR, doripenem; E, erythromycin; IPM, imipenem; K, kanamycin; MEM, meropenem; OX, oxacillin; S, streptomycin; SXT, trimethoprim/sulphamethoxazole; TE, tetracycline; TGC, tigecycline; TIC, ticarcillin; TOB, tobramycin; TZP, piperacillin/tazobactam; VA, vancomycin.

### Plasmid-based complementation of resistance genes reveals individual contributions to AMR

As none of the antibiotic resistance genes have been genetically tested for their individual contributions towards antibiotic resistance in *A. baumannii* AB5075 except for the role of *cmlA* in resistance to chloramphenicol, we aimed to characterise seven predicted p1AB5075-encoded antibiotic resistance genes *aacA4* (*ABUW_RS19275*), *aadA2* (*ABUW_RS19305*), *aadB* (*ABUW_RS19315*), *adh* (*ABUW_RS19420*), *strA* (*ABUW_RS19300*), *strB* (*ABUW_RS19295*) and *bla_GES-11_* (*ABUW_RS19280*) by ectopically expressing them from a plasmid in the Δp1AB5075 strain. Plasmid-based complementation in *A. baumannii* routinely utilizes the shuttle plasmid (or its origin of replication) pWH1266, which has been assembled from pBR322 and pWH1277. The latter pWH1277 is only partially sequenced; therefore, we first sequenced the plasmid pWH1266, which assembled into a plasmid of 8,911 bp.

**Fig. 3.**
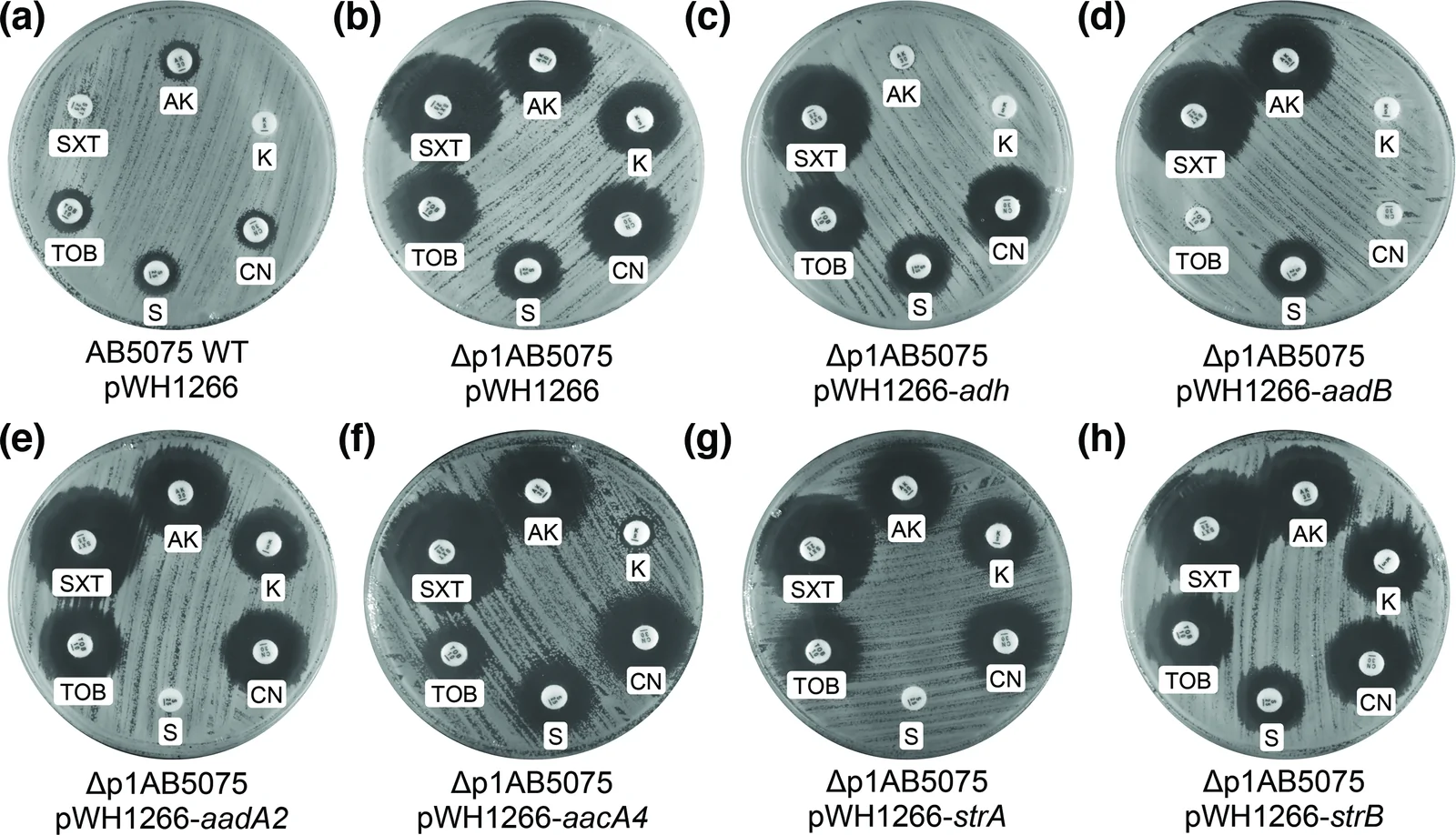
Antibiotic disc diffusion assays comparing *A. baumannii* AB5075 wild-type (WT, **a**) and Δp1AB5075 (**b**) strains carrying pWH1266 or pWH1266-ARG: pWH1266-*adh* (**c**), pWH1266-*aadB* (**d**), pWH1266-*aadA2* (**e**), pWH1266-*aacA4* (**f**), pWH1266-*strA* (**g**) or pWH1266-*strB* (**h**). AK (amikacin 30 µg), SXT (trimethoprim–sulphamethoxazole 25 µg), TOB (tobramycin 10 µg), S (streptomycin 25 µg), CN (gentamicin 30 µg) and K (kanamycin 5 µg). MH2 agar plates containing tetracycline were lawned with wild-type *A. baumannii* AB5075 and Δp1AB5075 and antibiotic-containing discs were placed on the agar surface. The plates were incubated for 24 h at 37 °C.

To compare resistance levels, all genes were expressed from the same pWH1266-endogenous promoter of the *blaTEM-1* gene (Fig. 1C) and equipped with the same ribosome binding site (AGGAGG) to ensure an equal rate of translation. During cloning, the *blaTEM-1* gene of pWH1266 is removed, leaving *tetA* as the sole (tetracycline) resistance gene. As controls, AB5075 WT and Δp1AB5075 were equipped with the pWH1266 ‘empty’ plasmid. None of the strains showed any severe growth defects when grown in l-broth (Fig. S1). Antibiotic disc diffusion assays were performed as before, and the phenotypes in WT and Δp1AB5075 strains remained the same in the presence of pWH1266 compared to the strains without pWH1266 (Figs 3, S2 and S3). As expected, mild resistance to tetracycline was acquired, which is mediated by *tetA* located on pWH1266 (Fig. S3). Typically, 12.5 µg ml^−1^ tetracycline is used in liquid broth; therefore, full resistance is not achieved to the 30 µg of the antibiotic-containing disc.

**Fig. 4.**
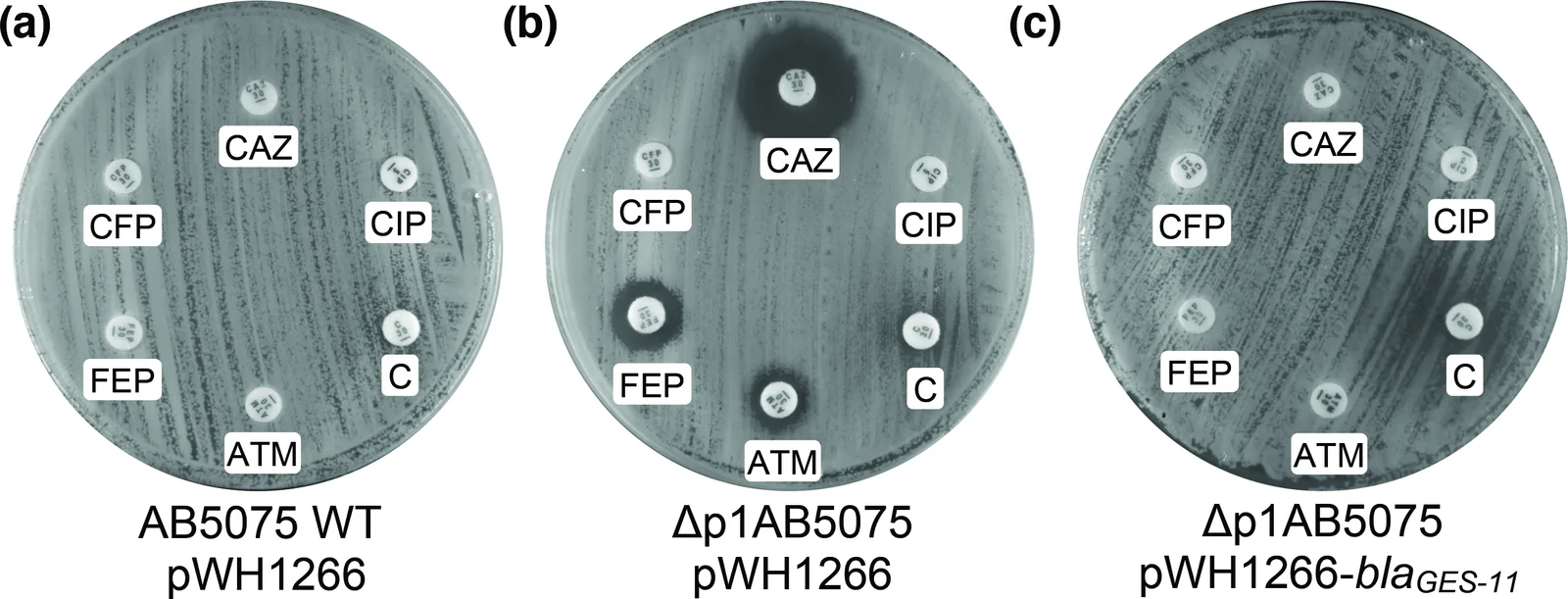
Antibiotic disc diffusion assays comparing *A. baumannii* AB5075 wild-type (WT, **a**) and Δp1AB5075 (**b**) strains carrying pWH1266 or pWH1266-*bla_GES-11_* (**c**). CAZ (ceftazidime, 30 µg), CFP (cefoperazone, 30 µg), FEP (cefepime, 30 µg), ATM (aztreonam, 30 µg), C (chloramphenicol, 30 µg), CIP (ciprofloxacin, 5 µg). MH2 agar plates containing tetracycline were lawned with wild-type *A. baumannii* AB5075 and Δp1AB5075 and antibiotic-containing discs were placed on the agar surface. The plates were incubated for 24 h at 37 °C.

Increased aminoglycoside susceptibility was noted upon deletion of p1AB5075 (Figs 2 and 3), and resistance could be restored by the expression of either *adh* (Fig. 3c), *aadB* (Fig. 3D), *aadA2* (Fig. 3e), *aacA4* (Fig. 3f) or *strA* (Fig. 3g). Expression of *adh* (APH(3′)-VI), which is not part of RI-2 and is flanked by two IS30 family transposases, restored resistance to kanamycin and further increased resistance to amikacin in comparison to the wild-type strain (Fig. 3a and c). Expression of *aadB* (ANT(2″)-Ia) restored resistance to kanamycin and increased resistance to gentamicin and tobramycin (Fig. 3a and d). Expression of *aacA4* only partially restored resistance to kanamycin (Fig. 3F) and *strA* provided resistance to streptomycin (Fig. 3g). Expression of *strB* did not restore resistance to any of the tested antibiotics (Fig. 3h). Therefore, the disc diffusion assays detected genes responsible for p1AB5075-mediated aminoglycoside resistance showing also overlap in providing resistance, where *adh*, *aadB* and to a lower extent *aacA4* restored resistance to kanamycin (Fig. 3c, d and f) and *aadA2* and *strA* provided resistance to streptomycin (Fig. 3e and g). Previously, *aacA4* was described as a potential pseudogene [9], we now show it is functional. The gene outside of RI-2, *adh*, was the only gene providing level resistance to amikacin, while RI-2-encoded *aadB* was the only gene restoring resistance to gentamicin and tobramycin. Expression of *bla*_GES-11_ restored resistance to three antibiotics: ceftazidime, cefepime and aztreonam (Fig. 4), which was in line with previous work [14].

To better quantify the contributions of the ARGs to antibiotic resistance and because there was overlap in providing resistance to several antibiotics, minimal inhibitory concentrations were determined by broth microdilution assays for selected antibiotics (Tables 3 and 4). No difference in the MIC was observed for seven clinically relevant antibiotics (Table 3). Expression of ARGs from pWH1266 not only restored resistance, but also routinely increased MICs, likely due to increased gene copy number and overexpression from the *blaTEM-1* promoter of the pWH1266 plasmid (Table 3). Expression of *aadB* increased the MIC to tobramycin from <0.5 to 128 µg ml^−1^ and to gentamicin from 8 to >256 µg ml^−1^, *adh* increased the MIC to amikacin from <4 to 512 µg ml^−1^, *aadA2* and *strA* increased the MIC to streptomycin from 16 to 1,024 µg ml^−1^ (Table 4) matching the data obtained from the disc diffusion assays (Fig. 2, Table 2). Complementation with pWH1266-*aacA4* restored the resistance to tobramycin to wild-type levels (16 µg ml^−1^) but also provided an increased low-level resistance to multiple aminoglycosides (increases of gentamicin 4-fold, kanamycin >32-fold and amikacin >2-fold) compared to Δp1AB5075 pWH1266 but did not reach wild-type resistance levels. As observed in disc diffusion assays, *strB* did not recover any of the antibiotic susceptibilities. Finally, the expression of *bla_GES-11_* increased resistance to cefepime (from 32 to 256 µg ml^−1^) and aztreonam (from 64 µg ml^−1^ to >256 µg ml^−1^), as shown in the disc diffusion experiments, but also showed increased resistance from 32 to 1,024 µg ml^−1^ to another tested cephalosporin (cefotaxime).

**Table 3.** Minimum inhibitory concentration of clinically relevant antibiotics for *A. baumannii* AB5075 determined by broth microdilution assay (*n*=4) Values are in μg ml^−1^.

Strain	CIP	NOR	LEV	TE	TGC	E	CS
WT	>32 (R)	> 256 (R)	2 (S)	0.5 (S)	<0.5 (S)	4	0.19 (S)
Δp1AB5075	>32 (R)	> 256 (R)	2 (S)	0.5 (S)	<0.5 (S)	4	0.19 (S)

*Organisms that are susceptible to tetracycline are also considered susceptible to doxycycline and minocycline.

CIP, ciprofloxacin; E, erythromycin; I, intermediate; LEV, levofloxacin; NOR, norfloxacin; R, resistant; S, susceptible; TE, tetracycline; TGC, tigecycline.

**Table 4.** MIC of antibiotics determined by broth microdilution assay (*n*=4) Values are in μg ml^−1^. CN (gentamicin), K (kanamycin), AK (amikacin), S (streptomycin), TOB (tobramycin), FEP (cefepime), CTX (cefotaxime), ATM (aztreonam), IPM (imipenem), MEM (meropenem), DOR (doripenem). nd (not determined). S (susceptible), I (intermediate), R (resistant).

Strain	CN	K	AK	S	TOB	FEP	CTX	ATM	IPM	MEM	DOR
WT pWH1266	>256 (R)	> 2048	128 (R)	128	16 (R)	>256 (R)	2048 (R)	>256	16 (R)	16 (R)	16
Δp1AB5075 pWH1266	8 (I)	<4	<4 (S)	16	<0.5 (S)	32 (R)	32 (I)	64	16 (R)	16 (R)	16
Δp1AB5075 pWH1266-*aadB*	> 256 (R)	512	<4 (S)	64	128 (R)	32 (R)	32 (I)	32	16 (R)	16 (R)	16
Δp1AB5075 pWH1266-*adh*	8 (I)	>2048	512 (R)	32	<0.5 (S)	32 (R)	32 (I)	64	16 (R)	16 (R)	16
Δp1AB5075 pWH1266-*aadA2*	8 (I)	<4	<4 (S)	1024	<0.5 (S)	32 (R)	32 (I)	32	16 (R)	16 (R)	16
Δp1AB5075 pWH1266-*aacA4*	32 (R)	128	8 (S)	32	16 (R)	32 (R)	32 (I)	32	16 (R)	16 (R)	16
Δp1AB5075 pWH1266-*strA*	8 (I)	<4	<4 (S)	1024	<0.5 (S)	32 (R)	32 (I)	32	16 (R)	16 (R)	16
Δp1AB5075 pWH1266-*strB*	8 (I)	<4	<4 (S)	32	<0.5 (S)	16 (I)	32 (I)	32	16 (R)	16 (R)	16
Δp1AB5075 pWH1266-*bla_GES-11_*	nd	nd	nd	nd	nd	256 (R)	1024 (R)	>256	16 (R)	16 (R)	16

## Discussion

Multidrug resistance in *A. baumannii* is mediated through extensive genetic diversity supported by different resistance islands, plasmids, including p1AB5075, and other mobile genetic elements. Here, we characterized the contribution of p1AB5075 and nine p1AB5075-encoded ARGs towards antibiotic resistance. We observed that loss of p1AB5075 renders *A. baumannii* AB5075 susceptible to multiple antibiotics including aminoglycosides, cephalosporins, trimethoprim/sulphamethoxazole and chloramphenicol. We used this Δp1AB5075 genetic background to genetically dissect the contributions of p1AB5075-encoded antibiotic resistance genes.

As the plasmid p1AB5075 is transferable from AB5075 to many other *A. baumannii* and non-*baumannii* strains, transfer would convert any antibiotic-susceptible strain to an MDR strain [10, 11]. We determined that amikacin resistance is predominantly mediated by a gene outside of RI-2 (*adh*, (*ABUW_RS19420*, APH(3′)-VI), which is flanked by IS30 family transposases, which have been described to contain ARGs in *A. baumannii* including the first description of a *bla*_NDM-1_ in Poland [29]. However, overexpression of *aacA4* increased the MIC to amikacin fourfold, showing that RI-2 amplification through recombination alone can lead to substantial amikacin resistance [13]. Indeed, overexpression of *aacA4* conferred low-level cross-resistance to other aminoglycosides (gentamicin, kanamycin and streptomycin) as well, expanding our knowledge of *A. baumannii* aminoglycoside resistance. Chloramphenicol resistance remained unchanged in the crude disc diffusion assay upon deletion of p1AB5075, which is in agreement with a previous study [17], and is due to the presence of additional chloramphenicol resistance genes *craA* and *cpxE* [30, 31].

To our knowledge, although expected upon loss of *bla_GES-11_*, the susceptibility to cephalosporins has not been shown for AB5075 yet. We now confirm experimentally that this is due to the presence of *bla*_GES-11_ located in RI-2. AB5075 Δp1AB5075 remains resistant to carbapenems imipenem and meropenem, though, due to the presence of a *blaOXA-23* gene on the chromosome. Similar to AB5075, a clinical isolate of *A. baumannii* from Tunisia with a chromosomally encoded *blaOXA-23* and a plasmid-encoded *bla*_GES-11_ copy isolated from a clinical sample was reported in 2014 [32]. Conjugation of the plasmid carrying *bla*_GES-11_ led to increased resistance to ticarcillin, ticarcillin/clavulanic acid, piperacillin, piperacillin/tazobactam, cefotaxime, ceftazidime, cefepime, aztreonam and even a slight increase in meropenem and imipenem, which might have been masked by the presence of *blaOXA-23* in our strains [14]. A concerning finding was that *bla*_GES-11_ may mutate (Gly170Ser) to *bla*_GES-14_, resulting in a significant increase in imipenem (from two to >32 µg ml^−1^) and meropenem (from 4 to 32 µg ml^−1^) resistance, as shown in *A. baumannii* isolated from wound infections of soldiers of Ukraine [33].

Apart from the clear role of pAB5075 for AMR in AB5075, future studies may investigate the wider impact on *A. baumannii* AB5075 biology as the plasmid is transferable. Other examples have been reported where (loss) of plasmids had profound effects on gene regulation and pathogenesis. The plasmid pAB3 was shown to repress type-6 secretion in *A. baumannii* ATCC17978 [16] and a plasmid-encoded copy of H-NS located on plasmid pAB5 in *A. baumannii* UPAB1 was reported to regulate biofilm formation [34]. As p1AB5075 also contains a copy of an H-NS family protein, there is potential that it might mediate similar crosstalk between p1AB5075- and chromosomally encoded genes. Certainly, a more comprehensive study of plasmids in *A. baumannii* will be required to better understand their impact on *A. baumannii* biology beyond AMR.

## Supplementary material

10.1099/jmm.0.002189 Supplementary Material 1.

## Abbreviations

AMR: antimicrobial resistance
ARGs: antibiotic resistance genes
MDR: multidrug-resistant
RI-2: Resistance Island 2
SLiCE: seamless ligation-independent cloning extract
