# Supplementary material for: Contributions of plasmid p1AB5075-encoded antibiotic resistance genes to multidrug resistance of Acinetobacter baumannii AB5075
Source: J Med Microbiol. 2026 Jul 24;75(7):002189. doi: 10.1099/jmm.0.002189 (PMC13405486; doi:10.1099/jmm.0.002189)
Supplement: Supplementary Material 1. [file jmm-75-02189-s001.pdf]

Supplementary Material

Contributions of plasmid p1AB5075-encoded antibiotic resistance genes to multidrug resistance of Acinetobacter baumannii AB5075

Orlaith Plunkett[1], Anna S. Ershova[1], Kristina Schauer[1] and Carsten Kröger[#,1]

[1]Department of Microbiology, School of Genetics & Microbiology, Moyne Institute of Preventive Medicine, Trinity College Dublin, Dublin 2, Ireland.

Table S1. Oligonucleotides used in this study. Red sequences indicate sequences homologous to ends of linearised pWH1266. Purple, underlined sequences indicate ribosome binding site sequence.

Name	Sequence (5′-3′)	Purpose
pWH1266_SLiCE_F	TATTGAAGCATTTATCAGGG	Amplification of pWH1266 backbone
pWH1266_SLiCE_R	CCTCACTGATTAAGCATTGG	Amplification of pWH1266 backbone
aac(6')-Ib3 F bla-pWH1266	CCAATGCTTAATCAGTGAGGCAGGGCCATTACCGATTACGCC	Amplification of *aac(6')-Ib3*
aac(6')-Ib3 R bla-pWH1266	CCCTGATAAATGCTTCAATAAGGAGGGCATCGTGACCAACAGCAACG	Amplification of *aac(6')-Ib3*
aph(3")-lb (StrA) F bla-pWH1266	CCAATGCTTAATCAGTGAGGGTCTTCTATAGGTTTCAATCCC	Amplification of *aph(3")-lb* (*strA*)
aph(3")-lb (StrA) R bla-pWH1266	CCCTGATAAATGCTTCAATAAGGAGGCTCCATTGAATCGGACTTATAT	Amplification of *aph(3")-lb* (*strA*)
aph(6)-l (StrB) F bla-pWH1266	CCAATGCTTAATCAGTGAGGCCGCTGCTATAGGGGTC	Amplification of *aph(6)-l* (*strB*)
aph(6)-l (StrB) R bla-pWH1266	CCCTGATAAATGCTTCAATAAGGAGGGGTTGATGTTCATGCCGC	Amplification of *aph(6)-l* (*strB*)
aadA1 F bla-pWH1266	CCAATGCTTAATCAGTGAGGCCACGTCGAAAAACAAAATCAC	Amplification of *aadA1*
aadA1 R bla-pWH1266	CCCTGATAAATGCTTCAATAAGGAGGGACATCATGAGGGTAGCGG	Amplification of *aadA1*
aadB F bla-pWH1266	CCAATGCTTAATCAGTGAGGCTGCTGGCTATCTCATGATTG	Amplification of *aadB*
aadB R bla-pWH1266	CCCTGATAAATGCTTCAATAAGGAGGGCCGCATGGACACAAC	Amplification of *aadB*
aph(3')-VI F bla-pWH1266	CCAATGCTTAATCAGTGAGGCTCAAGCATTAATGCAGTACGATC	Amplification of *aph(3')-VI*
aph(3')-VI R *bla*-pWH1266	CCCTGATAAATGCTTCAATAAGGAGGACTTGATGGAATTGCCCAATATTA	Amplification of *aph(3')-VI*
bla_{GES} *11* F *bla*-pWH1266	CCAATGCTTAATCAGTGAGGGCCTGAGTTAAGCCGCGGTGC	Amplification of bla_{GES} *11*
bla_{GES} *11* R *bla*-pWH1266	CCCTGATAAATGCTTCAATAAGGAGGTCACCATGCGCTTCATTCACGCAC	Amplification of bla_{GES} *11*

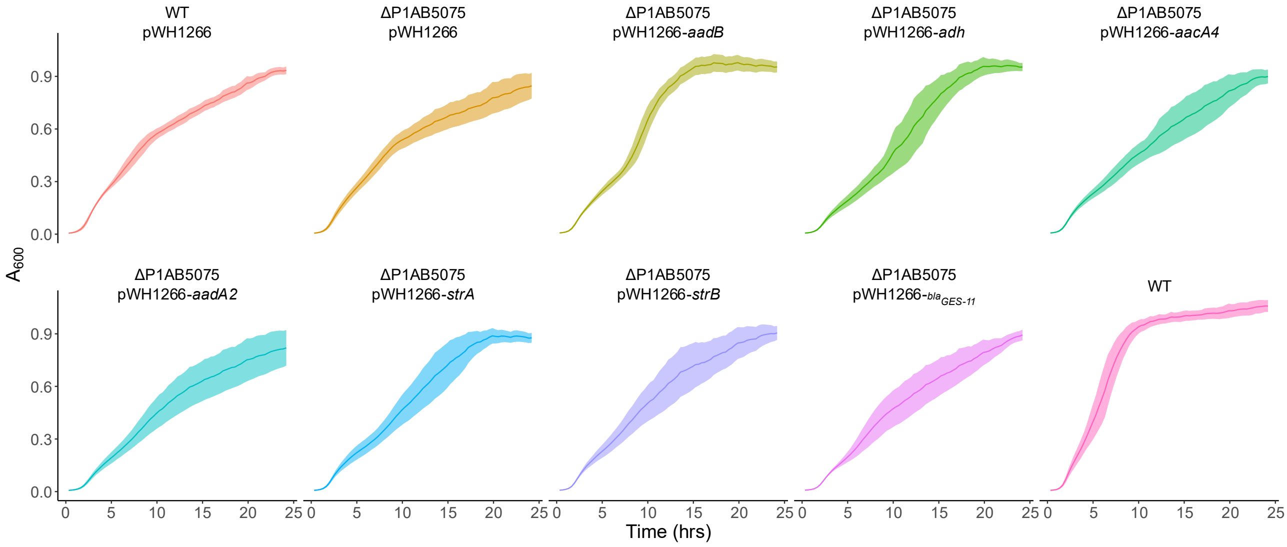

Supplementary Figure 1: Growth of *A. baumannii* AB5075 strains in L-broth containing tetracycline and harbouring the depicted plasmids. The strain labelled WT is the *A. baumannii* AB5075 wild-type strain grown in L-broth without tetracycline. Growth experiments were carried out in 96-well plates. The solid line depicts the mean value from eight biological replicates, and the lighter colour shows the standard deviation.

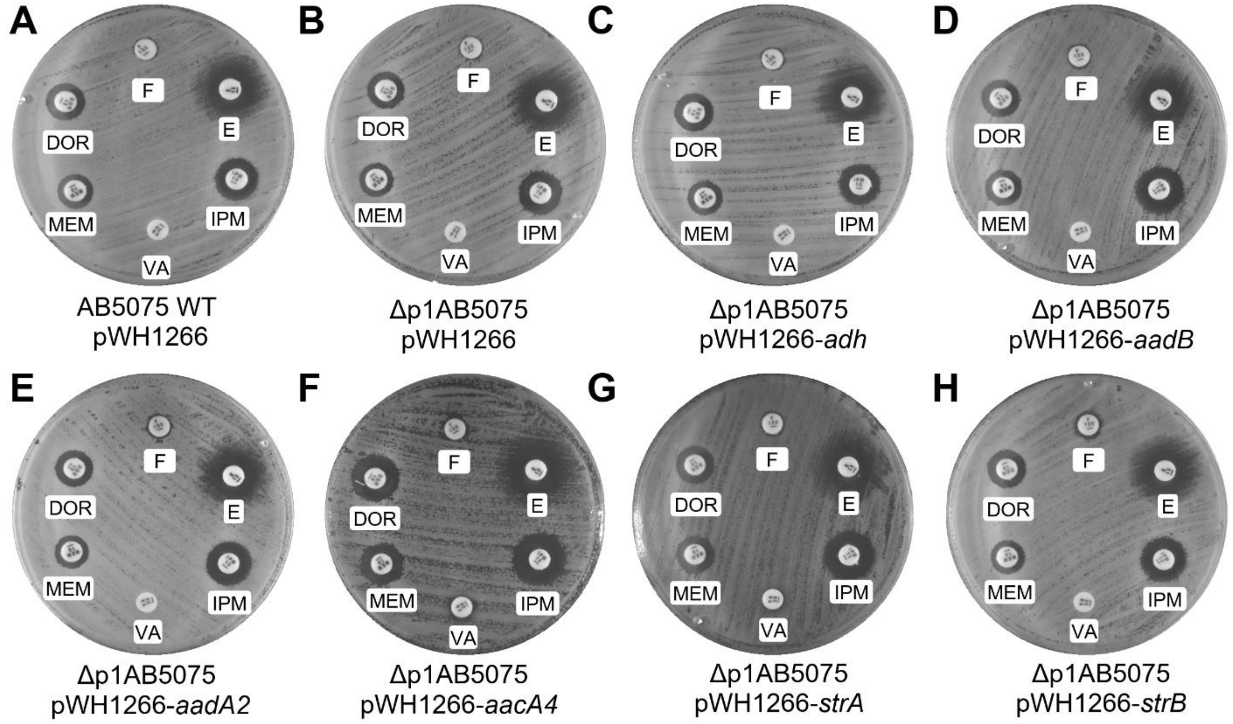

Supplementary Figure 2: Antibiotic disk diffusion assays comparing *A. baumannii* AB5075 wild-type (WT, A) and Δp1AB5075 (B) strains carrying pWH1266 or pWH1266-ARG: pWH1266-*adh* (C), pWH1266-*aadB* (D), pWH1266-*aadA2* (E), pWH1266-*aacA4* (F), pWH1266-*strA* (G) or pWH1266-*strB* (H). Nitrofurantoin (F, 100 µg), erythromycin (E, 15 µg), imipenem (IMP 10 µg), vancomycin (VA, 30 µg), meropenem (MEM, 10 µg), doripenem (DOR, 10 µg). MH2 agar plates containing tetracycline were lawned with wild-type *A. baumannii* AB5075 and Δp1AB5075 and antibiotic-containing disks were placed on the agar surface. The plates were incubated for 24 h at 37°C.

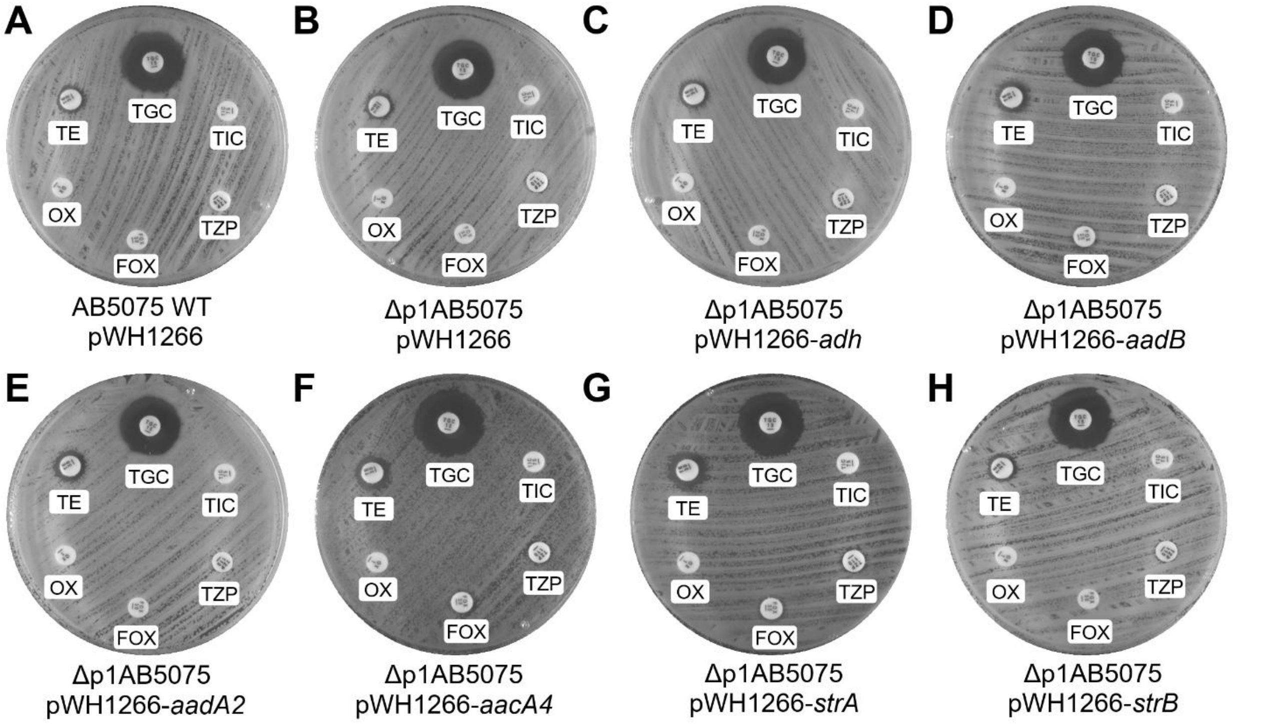

Supplementary Figure 3: Antibiotic disk diffusion assays comparing *A. baumannii* AB5075 wild-type (WT, A) and Δp1AB5075 (B) strains carrying pWH1266 or pWH1266-ARG: pWH1266-*adh* (C), pWH1266-*aadB* (D), pWH1266-*aadA2* (E), pWH1266-*aacA4* (F), pWH1266-*strA* (G) or pWH1266-*strB* (H). Tigecycline (TGC 15 µg), ticarcillin (TIC, 75 µg), piperacillin/tazobactam (TZP, 110 µg), cefoxitin (FOX, 30 µg), oxacillin (OX, 1 µg), tetracycline (TE, 30 µg). MH2 agar plates containing tetracycline were lawned with wild-type *A. baumannii* AB5075 and Δp1AB5075 and antibiotic-containing disks were placed on the agar surface. The plates were incubated for 24 h at 37°C.
